# Effects of a home-based activation intervention on self-management adherence and readmission in rural heart failure patients: the PATCH randomized controlled trial

**DOI:** 10.1186/s12872-016-0339-7

**Published:** 2016-09-08

**Authors:** Lufei Young, Melody Hertzog, Susan Barnason

**Affiliations:** 1 Department of Physiological and Technological Nursing, College of Nursing Augusta University, 987 St. Sebastian Way, Augusta, GA 30912 USA; 2 University of Nebraska Medical Center, College of Nursing, Omaha, Nebraska USA

## Abstract

**Background:**

Heart failure (HF) patients discharged from rural hospitals have higher 30-day readmission rates. Self-management (SM) reduces readmissions, but adherence to SM guidelines is low in the rural HF population. We tested a home-based intervention to enhance patient activation and lead to improved SM adherence.

**Methods:**

In this two-group, repeated measures randomized control trial, the main outcomes were patient reported and clinical outcomes associated with SM adherence, and all-cause readmission at 30, 90 and 180 days.

**Results:**

The study included 100 HF patients discharged from a rural critical access hospital. The intervention group received a 12-week SM training and coaching program delivered by telephone and tailored on subjects’ activation levels. At α = .10, the PATCH intervention showed significantly greater improvement compared to usual care in patient-reported SM adherence: weighing themselves, following a low-sodium diet, taking prescribed medication, and exercising daily (all *p* < .0005) at 3 and 6 months after discharge. In contrast, groups did not differ in physical activity assessed by actigraphy or in clinical biomarkers. Contrary to expectation, the 30-day readmission rate was significantly higher (*p* = .088) in the intervention group (19.6 %) than in the control group (6.1 %), with no differences at 90 or 180 days.

**Conclusion:**

It is feasible to conduct a randomized controlled trial in HF patients discharged from rural critical access hospitals. Significantly higher patient-reported SM adherence was not accompanied by lower clinical biomarkers or readmission rates. Further research is needed to understand mechanisms that influence outcomes and healthcare utilization in this population.

**Trial registration:**

Clinical Trial Registration Information: ClinicalTrials.gov; NCT01964053.

## Background

Compared to their urban counterparts, HF patients discharged from rural hospitals, primarily critical access hospitals (CAHs), have higher 30-day readmission [1]. Failing to adhere to self-management (SM) guidelines accounted for 50 % of hospital readmissions in HF patients [2]. SM adherence involves engaging in recommended SM behaviors such as monitoring daily weight, following a restricted sodium diet, taking medication as prescribed, exercising regularly, and keeping follow-up appointments [3].

To date, most strategies promoting SM adherence were developed and tested in urban health centers [4]. Less is known about these interventions’ feasibility and efficacy in rural areas where resources such as HF specialists and multidisciplinary HF management teams are often limited [5]. Further, rural patients may have lower self-management knowledge and health literacy [5], lack of HF-specific SM education and counselling from providers [6], and lack of SM support [7]. Consequently, rural HF patients tend to exhibit low SM adherence [5]. Past research also has been limited by lack of a theoretical framework [8], unclear mechanism of the intervention [9], and lack of objective measures of SM adherence [10].

To address the knowledge gap, we conducted a randomized controlled trial to test an activation-enhancing intervention to improve SM adherence. Patient activation is defined by Hibbard as the person’s readiness, willingness, and ability to manage his/her own health and healthcare [11]. We hypothesized that the HF patient with higher activation will have greater SM adherence. The purpose of this study is to examine the effects of a 12-week patient activation intervention (**P**atient **A**c**T**ivated **C**are at **H**ome [PATCH]) on the improvement of SM adherence and its health outcome (i.e., hospital readmission) in HF patients following discharge from CAHs.

This study had the following specific aims:

- Aim 1. To evaluate the immediate (3 months) and extended effects (6 months) of the patient activation intervention on SM adherence.
- Aim 2. To evaluate the effects of the patient activation intervention on hospital readmission and emergency department (ED) visit rates at 30 days, 3 and 6 months.
- Aim 3. To evaluate the mechanism of the patient activation intervention, comparing the intervention and usual care (UC) groups on SM knowledge, self-efficacy for SM, patient activation, and SM strategies at the end of intervention.

## Conceptual framework

To guide the PATCH intervention, we developed a conceptual framework based on components of Lorig’s chronic disease self-management model [12], Hibbard’s patient activation theory [13], and Bandura’s conceptualization of self-efficacy [14]. The mechanism of intervention is to improve HF patients’ SM adherence by helping them advance through four activation levels: 1) starting to take a SM role; 2) building SM knowledge, skills and confidence; 3) taking SM actions; and 4) maintaining SM behaviors. The central hypothesis is that HF patients with higher levels of activation will be more likely to engage in SM behaviors, leading to improved clinical biomarkers and fewer hospital readmissions [15].

## Methods

### Study design

This was a two-group, repeated measures, randomized controlled trial. The study is registered on the Clinical Trial website (NCT01964053). The study protocol was approved by the University of Nebraska Medical Center Institutional Review Board and rural hospital ethics committee. All participants gave written informed consent. Detailed information has appeared in a previously published study protocol [16].

#### Study setting

The study was conducted between September 2013 and October 2015 at a rural critical access hospital (CAH). To reduce the financial vulnerability of rural hospitals and improve rural residents’ healthcare access, Centers for Medicare and Medicaid Services (CMS) created a “Critical Access Hospitals (CAHs)” designation based on the 1997 Balanced Budget Act. A certified rural CAH must have less than 25 acute care inpatient beds and be located more than 35 miles from another hospital [17].

### Patient inclusion and exclusion

The principal investigator and research assistants who have ethical access at the study site were responsible for identifying potential subjects, screening for eligibility and recruitment. Eligible subjects: 1) were 21 or older; 2) had HF as one of their discharge diagnoses; 3) had New York Heart Association (NYHA) class II to IV symptoms or NYHA class I symptoms and at least one other HF-related hospitalization or ED visit in the previous year; 4) were discharged to home; 5) passed a mini-cog screen;[18] 6) understood English; and 7) had access to a phone. Patients were not eligible if they had: 1) scheduled procedures/surgeries during hospitalization; 2) depressive symptoms indicated by a score ≥ 3 on the Patient Health Questionnaire-2 (PHQ-2);[19] 3) documented diagnostic evidence of liver cirrhosis; or 4) renal failure (serum creatinine greater than 2.0 mg/dl).

### Intervention and usual care

Subjects randomized to the Control Group received only usual care, the standard discharge teaching for HF that includes written and verbal information about HF self-care and scheduled follow-up doctor appointments. Subjects randomized to the Intervention Group received both usual care and the 12-week PATCH intervention. The intervention was comprised of two phases: a one-on-one in-hospital SM training session and post-discharge reinforcement sessions (twice a week for the first 2 weeks, once a week for weeks 3–6, and every other week for weeks 7–12) delivered by telephone. Intervention content was presented in a variety of formats (e.g., verbal, written, visual) with interactive ability. Besides SM workbooks, each subject was provided an SM toolkit, including a calendar for weight and salt daily logging, a step-on weight scale with large and bright readings, and an electronic pill organizer reminder alarm. Each intervention session lasted about 45–50 min. Booster sessions were administered to subjects struggling with SM at home. Subjects received the tailored intervention sessions based on activation level, pre-set goals, and specific SM needs. Intervention details were reported in another publication [16].

### Outcome measures

Baseline data collection occurred prior to hospital discharge and at 3 and 6 months after discharge. The primary outcomes measured at all three times were: SM adherence (self-reported frequencies of daily weighing, following a low-sodium diet, taking prescribed medications, exercising, and attending follow-up appointments), clinical biomarkers (B-type natriuretic peptid**e** [BNP] and urine sodium/creatinine ratio [Na/Cr]), and all-cause readmissions and ED visits measured at 30, 90 and 180 days. To assess baseline SM adherence, we asked the participants to recall specific SM behaviors in the past 12 months. The healthcare utilization data were collected from both self-report and primary care provider records. In addition to self-report measures, objective measures of physical activity were obtained using an accelerometer that subjects were asked to wear for 7 consecutive days at each assessment period.

The secondary outcomes were measured via questionnaire at baseline and 3 months to test the intervention mechanisms, including SM knowledge, self-efficacy for SM, patient activation, and SM strategies. Details about measures, instruments and their psychometric properties were reported in another publications [16, 20, 21].

### Randomization, blinding and allocation concealment

Given the nature of the treatment, blinding of either subject or interventionist was impossible, but the data collector was blinded to treatment assignment. The project statistician used an on-line pseudo-random number generator to create an allocation schedule; random ordering of block sizes four and six was used to maintain even accrual through the study. Group assignments were placed in sealed envelopes and opened sequentially as patients were enrolled.

### Sample size and statistical analysis

The required sample size was estimated using two-sided tests and α = .10. A liberal alpha was chosen to minimize the likelihood of overlooking promising effects in this preliminary study. For a moderate effect size (Cohen’s f = .25), 41 per group provided power of .80 for the test of the mean group difference over time. It provided similar power for a z-test of the difference in group proportions of at least .25. A target sample size of 100 patients allowed for up to 20 % attrition. Further details may be found in Young et al. [16].

For the continuous outcomes in Aim 1 (e.g., physical activity outcomes and level of BNP and urine Na/Cr), linear mixed model methods were used to compare the groups across the 6-month period, adjusting for baseline levels on the respective outcome, with tests of the difference in estimated marginal means (Group effect) and whether change from 3 to 6 months differed in the groups (Group X Time effect). These methods allow for inclusion of partial cases (missing either month 3 or month 6 follow-up data) and for flexible specification of the covariance structure of repeated measurements. However, cases missing on covariates or having only covariate (baseline) measures cannot be included.

Distributions of adherence outcomes measured as number of days per week were clearly non-normal, so responses to those questions also were categorized as non-adherent (0 days), partially adherent (1–6 days), or adherent (7 days), and groups compared using *χ*^2^. For outcomes having adherence guidelines (e.g., weighing), patients also were classified as being adherent or not and group differences in the proportion of adherent patients at the end of the intervention (3 months) and at 6 months were tested using *χ*^2^.

To evaluate immediate and extended effects of the intervention on rehospitalization and ED visits (Aim 2), a *χ*^2^ test was used to compare group proportions separately at 30 days, 3, and 6 months.

To evaluate the mechanism of the patient activation intervention (Aim 3), an independent *t*-test was used to compare the groups on average change in intervention components from baseline to 3 months after hospital discharge. These tests were one-tailed to correspond to the hypothesis that the PATCH intervention would increase SM knowledge, self-efficacy, activation levels, and use of SM strategies.

Effect sizes were also estimated for the estimated marginal mean difference between groups. There is no established method of estimating effect sizes in linear mixed models, so standardization (*d* = |M_1_ – M_2_| / SD) was carried out using the baseline standard deviation of the outcome, pooled across groups. For variables having no baseline measurement, the standard deviation from the control group at 3 months was used. For tests of intervention components, the group difference in mean change was standardized using a pooled standard deviation of the change scores.

## Results

**Fig. 1:**
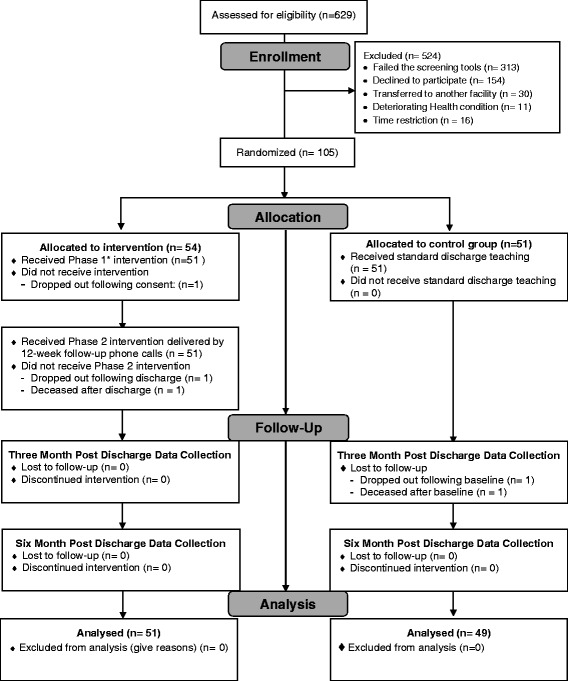
Study flow diagram

Between September 2013 and October 2015, 629 potentially eligible candidates were screened. Of these, 524 subjects were excluded because of 1) failing to meet the screening criteria; 2) declining participation; 3) being transferred to another facility during hospital stay; 4) deteriorated health conditions; or 5) less than 24-h length of stay, which made it impractical to conduct Phase I intervention and baseline data collection. After randomization, three subjects were excluded from the PATCH intervention group and two from the control group as described in the CONSORT study flow diagram (Fig. 1). A sample of 100 HF subjects (PATCH = 51, control = 49) entered the final analysis.

### Sample characteristics

The 64 females and 36 males ranged in age from 40 to 93, with a mean of 70.2 (±12.2) years. On average, they had 12.9 (±2.3) years of education and a median annual household income of $20,000–29,000. Most were NYHA level II (49 %) or III (42 %) with preserved ejection fraction (55.7 ± 11.1). On average, patients had eight (±2.6) comorbidities, with the overwhelming majority having hypertension (99 %), coronary artery disease (94 %), arthritis or degenerative joint disease (89 %), and hypercholesterolemia (84 %). Subjects reported having an average of 12.3 (±6.0) prescriptions, requiring 16.2 (±8.8) pills per day (Table 1). At hospital discharge, 50 % of the subjects were prescribed beta blockers and 52 % either ACE inhibitors or angiotensin receptor blockers.

**Table 1:** Patient demographic and clinical characteristics

	All (*n* =100)	Intervention group (*n* = 51)	Control group (*n* = 49)
Demographic data			
Age (years)	70.2 ± 12.2	68.7 ± 11.8	71.8 ± 12.6
Male	36 (36)	24 (47.1)	12 (24.5)
Education (years)	12.9 ± 2.3	13 ± 2.4	12.8 ± 2.1
Caucasian	95 (95)	48 (94.1)	47 (95.9)
Married/living with partner	50 (50)	31 (60.8)	19 (38.8)
Currently employed outside home	29 (29)	16 (30.8)	13 (26.5)
Annual family income (< $30,000)	51 (51)	24 (47.10)	27 (55.1)
Risk factor profile			
Body Mass Index (kg/m^2^)	32.3 ± 7.1	33.4 ± 7.4	31.2 ± 6.8
Clinical data			
Number of comorbidities	8 ± 2.6	7.8 ± 2.5	8.0 ± 2.7
1.Hypertension	99 (99)	51 (100.0)	48 (98.0)
2.Coronary artery disease	94 (94)	46 (90.2)	48 (98)
3.Arthritis degenerative joint disease	89 (89)	43 (84.3)	44 (89.8)
4.Hypercholesterolemia	84 (84)	43 (84.3)	41 (83.7)
5.Diabetes mellitus with or without complications	41 (41)	41 (80.4)	33 (67.4)
6.Dyspepsia	50 (50)	24 (47.1)	26 (53.1)
7.Peripheral vascular disease or lower extremity edema	45 (45)	22 (43.1)	23 (46.9)
8.Chronic obstructive pulmonary disease	38 (38)	22 (43.1)	16 (32.7)
9.Chronic renal disease	23 (23)	12 (23.5)	11 (22.4)
Number of medications taking per day	16.2 (±8.8)	16.4 ± 10.0	15.9 ± 7.4
Beta blockers	50 (50)	27 (52.9)	23 (46.9)
ACE inhibitors	35 (35)	16 (31.4)	19 (38.8)
Angiotensin receptor blocker	18 (18)	8 (15.7)	10 (20.4)
Previous hospitalizations within 1 year (≥1)	43 (43)	22 (43.2)	21 (42.8)
Previous ED visits within 1 year (≥1)	60 (60)	30 (58.9)	30 (61.2)
Cardiac function			
Functional class (NYHA)			
•II	49 (49)	15 (29.4)	34 (69.4)
•III	42 (42)	29 (56.9)	13 (26.5)
Ejection fraction^a^	55.7 ± 11.1	53.4 ± 12.9	58.3 ± 8.1
Ejection fraction < 50 %^a^	16 (16)	12 (23.5)	4 (8.2)
Main outcome: Patient Activation Measure (PAM) level			
1.Starting to take a role	39 (39)	19 (37.3)	20 (40.8)
2.Building knowledge, skills and confidence	23 (23)	9 (17.6)	14 (28.6)
3.Taking action	18 (18)	13 (25.5)	5 (10.2)
4.Maintaining behaviors	19 (19)	10 (19.6)	9 (18.4)

Results are presented as mean ± SD or n (%)

^a^Ejection fraction was available for *n* = 47 in the intervention group, *n* = 41 in the usual care group. Percentages include cases with missing data in the denominator

The PATCH intervention and control groups were comparable on all demographic and baseline clinical characteristics with a few exceptions. A detailed breakdown is presented in Table 1. There were more females in the control group (75.5 %) than in the intervention group (52.9 %); only 38.8 % were married or partnered in the control group compared to 60.8 % in the intervention group. Those randomized to the intervention group had lower cardiac function than those in control group. Compared to subjects in the control group (26.5 %), more than twice as many subjects (56.9 %) in the intervention group were NYHA class III with ejection fraction less than 50 (23.5 % in PATCH group vs. 8.2 % in control group).

At baseline, the majority of subjects had low activation in terms of SM adherence. Patient activation measure (PAM) scores placed 39 % of subjects in activation level 1 (starting to take a role), 23 % in level 2 (building knowledge, skills and confidence), 18 % in level 3 (taking action), and 19 % in level 4 (maintaining behaviors).

- Aim 1. To evaluate the immediate and extended effects of the patient activation intervention on SM adherence.

Descriptive statistics for SM adherence outcomes and the results of the statistical tests from fitting a linear mixed model are presented in Table 2. None of the tests of Group x Time interaction were significant. Those receiving the PATCH intervention had significantly higher self-reported adherence to SM behaviors, including average days per week weighing themselves (*p* < .0005; *d* = 1.1), following a low-sodium diet (*p* < .0005; *d* = .9), and exercising (*p* < .0005; *d* = .6). The intervention group also reported significantly fewer days missing any doses of prescribed medication (*p* = .030; *d* = .6) (Table 2). Approximately twice as many patients in the intervention group as in the control group reported that they carried out these behaviors 7 days a week. Details of the frequencies of SM adherence for these outcomes at 3 and 6 months are displayed in Table 3. In the intervention group, approximately 84 % at 3 months and 86 % at 6 months reported not missing any doses in the previous week compared to 68 % at both times in the control group. Nearly all subjects (100 % of the PATCH group, 98 % of the control group) attended the scheduled follow-up appointment with their primary care provider within 30 days of discharge.

**Table 2:** Self-management adherence outcomes by group and time

Variable	Group	Unadjusted Mean^a^ (SD)		Estimated^b^ Marginal Mean (SE)	Group (*p*-value)	Group X Time (*p*-value)	Estimated group difference in marginal means [95 % CI]
		3 months	6 months				
Adherence to self-management behaviors per self-report							
Days per week weigh self	Intervention	4.8 ± 2.7	4.6 ± 2.4	4.7 ± 0.3	<.0005	.687	2.98 [2.10, 3.86]
	Control	1.9 ± 2.7	1.5 ± 2.5	1.7 ± 0.3			
Days per week follow low-sodium diet	Intervention	5.6 ± 2.1	5.1 ± 2.6	5.3 ± 0.3	<.0005	.668	2.62 [1.74, 3.50]
	Control	3.1 ± 2.9	2.3 ± 3.0	2.7 ± 0.3			
Days missed any medication doses in past 7 days	Intervention	.39 ± 1.2	.26 ± 1.0	0.3 ± 0.2	.030	.544	−0.51 [−0.97, −0.05]
	Control	.81 ± 1.6	.83 ± 1.4	0.8 ± 0.2			
Days per week exercise	Intervention	5.4 ± 1.8	4.5 ± 2.5	4.9 ± 0.3	<.0005	.230	1.66 [0.79, 2.53]
	Control	3.4 ± 2.9	3.1 ± 2.5	3.3 ± 0.3			
Physical Activity measured by ActiGraph^c^							
Average daily activity counts	Intervention	285,707 ± 152,860	306,648 ± 165,316	279,160 ± 19,821	.780	.693	8236 [−50,155; 66,628]
	Control	251,265 ± 145,744	250,913 ± 275,411	270,924 ± 271,313			
Average activity kcals/kg/day	Intervention	2.2 ± 1.6	2.3 ± 1.9	2.08 ± 0.2	.773	.664	0.07 [−0.42, 0.56]
	Control	1.8 ± 1.7	1.7 ± 1.8	2.01 ± 0.2			
Average daily mins ≥ moderate intensity activity	Intervention	5.8 ± 8.8	5.9 ± 10.8	4.81 ± 0.9	.897	.860	0.17 [−2.34, 2.71]
	Control	3.3 ± 10.5	3.7 ± 7.1	4.65 ± 0.9			
Adherence to self-management behaviors measured by biomarkers							
B-type natriuretic peptide (log-transformed)	Intervention	1.7 ± 0.7	1.7 ± 0.6	1.70 ± 0.1	.282	.512	−0.09 [−0.26, 0.08]
	Control	1.8 ± 0.5	1.8 ± 0.4	1.80 ± 0.1			
Average daily sodium intake (<1500 mg)	Intervention	3607.0 ± 1395.3	3748.7 ± 1586.2	3647.9 ± 157.3	.234	.818	−272.03 [−722.70, 178.65]
	Control	3876.9 ± 1097.2	3926.6 ± 1279.3	3919.9 ± 163.5			

^a^ Observed means for the intervention group were based on *n* = 51, 51, and 50 cases for self-report measures, biomarkers, and actigraphy variables, respectively, at month 3 and *n* = 50, 50, and 47 at month 6. For the control group, *n* = 47, 47, and 46 at month 3 and *n* = 46–47, 47, and 47 at month 6. The supplemental analysis reported in the text that included baseline as a time point was based on all 99 cases having any actigraphy data at any time

^b^ Estimated marginal means and statistical tests were obtained from a linear mixed model analysis, adjusted for baseline values of the outcome and specifying an unstructured variance/covariance matrix

^c^ Valid time needed to assess physical activity by ActiGraph was a minimum of 8 h of wear time or at least 2 valid days were required for calculation of composites at each time point. The mean (± SD) days worn ranged from 5.9 (±1.4) to 6.3 (±1.2) days. Group mean hours worn ranged from 15.2 (±2.6) to 16.2 (±3.2)

**Table 3:** Frequencies of self-management adherence by outcomes, group and time

Variable	Group		Baseline	3 Months	6 Months
Days missed any medication doses in past 7 days^a^	Intervention	0 days	86.3 % (44/51)	84.3 % (43/51)	86.0 % (43/50)
		≥1 day	13.7 % (7/51)	15.7 % (8/51)	14.0 (7/50)
	Control	0 days	81.6 % (40/49)	68.1 % (32/47)	68.1 % (32/47)
		≥1 day	18.4 % (9/49)	31.9 % (15/47)	31.9 % (15/47)
			*χ* ^2^ = .130 (*p* = .719) ^b^	*χ* ^2^ = 2.74 (*p* = .098) ^b^	*χ* ^2^ = 3.47 (*p* = .060) ^b^
Kept scheduled appointment with primary care provider (within 30 days)	Intervention		100 % (51/51)		
	Control		97.9 % (46/47)		
			Fisher’s exact *p* = .295^c^		
Days/wk follow low-sodium diet	Intervention	0 days	-	7.8 % (4/51)	14.0 % (7/50)
		1 – 6 days	-	35.3 % (18/51)	30.0 % (15/50)
		7 days	-	56.9 % (29/51)	56.0 % (28/50)
	Control	0 days	-	38.3 % (18/47)	55.3 % (26/47)
		1 – 6 days	-	40.4 % (19/47)	21.3 % (10/47)
		7 days	-	21.3 % (10/47)	23.4 % (11.47)
				*χ* ^2^ = 18.06 (*p* < .0005)	*χ* ^2^ = 19.28 (*p* < .0005)
Days/wk exercise	Intervention	0 days	-	2.0 % (1/51)	12.0 % (6/50)
		1 – 6 days	-	52.9 % (27/51)	50.0 % (25/50)
		7 days	-	45.1 % (23/51)	38.0 % (19/50)
	Control	0 days	-	23.4 % (11/47)	23.9 % (11/46)
		1 – 6 days	-	42.6 % (20/47)	58.7 % (27/46)
		7 days	-	34.0 % (16/47)	17.4 % (8/46)
				*χ* ^2^ = 10.49 (*p* = .005)	*χ* ^2^ = 5.87 (*p* = .053)
Days/wk weigh self	Intervention	0 days	-	13.7 % (7/51)	6.0 % (3/50)
		1 – 6 days	-	41.2 % (21/51)	56.0 % (28/50)
		7 days	-	45.1 % (23/51)	38.0 % (19/50)
	Control	0 days	-	46.8 % (22/47)	53.2 % (25/47)
		1 – 6 days	-	34.0 % (16/47)	31.9 % (15/47)
		7 days	-	19.1 % (9/47)	14.9 % (7/47)
				*χ* ^2^ = 14.42 (*p* = .001)	*χ* ^2^ = 26.69 (*p* < .0005)
≤1500 mg Na daily	Intervention	Not met	96.1 % (49/51)	98.0 % (49/50)	98.0 % (49/50)
		Met	3.9 % (2/51)	2.0 % (1/51)	2.0 % (1/50)
	Control	Not met	100 % (49/49)	100 % (47/47)	100 % (47/47)
		Met	0 % (0/49)	0 % (0/47)	0 % (0/47)
			Fisher’s exact *p* = .495^c^	Fisher’s exact *p* = 1.00^c^	Fisher’s exact *p* = 1.00^c^
Exercise 7 days/wk (self-report)	Intervention	Not met	-	54.9 % (28/51)	62.0 % (31/50)
		Met	-	45.1 % (23/51)	38.0 % (19/50)
	Control	Not met	-	66.0 % (31/47)	82.6 % (38/46)
		Met	-	34.0 % (16/47)	17.4 % (8/46)
				*χ* ^2^ = 1.25 (*p* = .264)	*χ* ^2^ = 5.03 (*p* = .025)
≥120 mins/wk of moderate or more intense activity^d^	Intervention	Not met	91.8 % (45/49)	88.0 % (44/50)	91.5 % (43/47)
		Met	8.2 % (4/49)	12.0 % (6/50)	8.5 % (4/47)
	Control	Not met	100 % (42/42)	95.7 % (44/46)	95.7 % (45/47)
		Met	0 % (0/42)	4.3 % (2/46)	4.3 % (2/47)
			Fisher’s exact *p* = .121^c^	Fisher’s exact *p* = .271^c^	Fisher’s exact *p* = .677^c^

^a^With three categories, days with missed medication doses did not meet the assumptions of the *χ* ^2^ test. Because very few patients missed 7 days at any time point (1 in the intervention group at 3 months and at 6 months and 1 in the control group at 6 months), data from 1 to 6 days and 7 days were combined

^b^With continuity correction for 2 × 2 table

^c^Fisher’s exact test was used for 2 × 2 tables when assumptions of *χ* ^2^ test were not met

^d^Criteria of recommended 150 min/wk considered met if 80 % (120 min) of target achieved

In contrast to the self-report measures, there were no significant group differences in the estimated mean activity counts, energy expenditure, or minutes in moderate or more intense activity, with small effect sizes (*d* = .03 to *d* = .05). Results from a supplemental analysis of the actigraphy variables that treated baseline as a time point in order to include all partial cases were consistent with these findings, with no significant differences between groups in the pattern of change across time on any of the actigraphy variables (*p*-values ranged from .589 to .858 for the tests of Group X Time interactions). B-type natriuretic peptide (BNP) was log-transformed due to strong positive skewness. Neither log- transformed BNP nor estimated daily sodium intake based on the urine test differed significantly in the two groups. There were also no significant group differences in the proportion meeting SM adherence criteria of ≤ 1500 mg of sodium daily and of ≥ 120 min per week of moderate or higher intensity activity (adherence defined as 80 % of the recommended 150 min per week), either at the end of the intervention (3 months) or at the 6-month follow up. Few patients in either group met these criteria at any point in the study (Table 3).

- Aim 2. To evaluate the effects of the patient activation intervention on hospital readmission and ED rates at 30 days, 3 and 6 months.

Contrary to expectation, the 30-day hospital readmission rate was significantly higher (*χ*^2^ with continuity correction = 2.914, *p* = .088) in the PATCH group (*n* = 10, 19.6 %) than in the control group (*n* = 3, 6.1 %). At 90 days and at 180 days, the groups were not significantly different (*n* = 12, 23.5 % and *n* = 12, 24.5 %, respectively, at 90 days; *n* = 18, 35.3 % and *n* = 20, 40.8 % at 180 days). A similar pattern was seen with ED visits at 30 days (*n* = 6, 11.8 % and *n* = 1, 2.0 %); 90 days (*n* = 9, 17.6 %; and *n* = 5, 10.2 %); and 180 days (*n* = 12, 23.5 % and *n* = 11, 22.4 %), but none of the differences were significant.

- Aim 3. To evaluate the mechanism of the patient activation intervention, comparing the intervention and UC groups on SM knowledge, self-efficacy for self-management, patient activation, and SM strategies at the end of intervention (3 months).

As shown in Table 4, the intervention group, on average, showed significantly greater increases in self-efficacy for heart failure SM (*p* = .034; *d* = .4), SM strategies (*p* < .0005; *d* = 1.0), and patient activation scores (*p* = .069; *d* = .3). No group differences were found for SM knowledge, which changed approximately two points in each group.

**Table 4:** Descriptives and t-tests of mean change in intervention component measures

Variable	Possible score range	Group	Number	Baseline Mean (SD)	Number	Month 3 Mean (SD)	Mean change (SD)	*t*-test (*p*-value) ^a^
Self-management knowledge^b^	0–27	Intervention	51	21.1 (2.8)	51	23.1 (2.7)	2.0 (3.4)	.337
		Control	49	19.4 (3.0)	47	21.1 (2.5)	1.7 (2.6)	
Self-efficacy for HF self-management^c^	0–100	Intervention	51	44.9 (24.7)	51	59.7 (17.3)	14.8 (26.0)	.034
		Control	49	49.2 (22.9)	47	53.8 (24.0)	5.2 (25.5)	
Patient activation (PAM Rasch scaled scores) ^d^	0–100	Intervention	51	57.3 (19.2)	50	69.1 (16.7)	11.7 (21.3)	.069
		Control	48	56.6 (18.6)	47	61.4 (18.2)	5.7 (18.5)	
Self-management strategies (RSCB) ^e^	0–145	Intervention	50	86.8 (19.3)	51	115.7 (19.6)	28.9 (23.0)	<.0005
		Control	49	91.9 (19.9)	47	97.6 (22.6)	5.6 (22.3)	

^a^One-tailed test of group difference in change from baseline to 3 months, equal variances not assumed

^b^Possible range for Self-management knowledge: 0-27

^c^Possible range for self-efficacy for HF self-management: 0-100

^d^Possible range for patient activation measure: 0-100

^e^Possible range for self-management strategies: 0-145

## Discussion

To our knowledge, this is the first efficacy trial of an activation-enhancing in rural HF patients. Similar to Shively’s and Hibbard’s studies [22, 23], the findings demonstrate that the theory-based activation-enhancing intervention was effective in increasing patient activation level, leading to improved SM behaviors in rural HF patients.

This study contributes to the field of SM in rural HF population by adding the following evidence: 1) understanding the effect of patient activation on SM behaviors, 2) development of a conceptual framework to guide the design and implementation of activation-enhancing interventions to promote life-long SM adherence; 3) using clinical biomarkers (BNP and urine Na/Cr) to assess SM adherence; and 4) feasibility and patients’ acceptance of home- based interventions to improve SM behaviors.

Based on self-reported data, HF patients in the PATCH intervention group had significant improvement in activation scores and SM adherence in weighing themselves daily, following a low-sodium diet and exercising regularly. According to the objective measures (i.e., physical activity measured by accelerometer and daily sodium intake computed from urine sodium), however, the subjects from the intervention group did not reach adherence threshold of sodium restriction and exercise intensity guidelines. Consequently, the reported improvement in SM adherence and behaviors did not improve clinical biomarkers or reduce readmissions. Instead, the 30-day readmission rate was higher in the intervention group than in the control group. Furthermore, the PATCH intervention had no impact on SM knowledge at 3 months.

Similar to the rural HF populations in other studies [24], our sample was predominantly white, retired, and low income. However, our study did have more female participants than have been reported in other studies. During recruitment, we found women more willing than men to participate in the study [15, 25, 26]. Field notes indicated women were more likely to believe SM had positive effects on their sustained independence and HF symptom relief, felt more isolated and more often sought social interactions with the research team, and had greater interest in learning SM strategies. Consistent with Dracup and Powell’s reports [5, 27], improved SM adherence through education and training did not lead to reduced readmissions in rural patients with mild to moderate heart failure.

There are several explanations for this deviation from mainstream belief about the benefit of SM on HF outcomes [28, 29]. First, low SM capability made it difficult and sometimes impossible for some HF patients to perform SM behaviors. SM capability is defined as the capability to manage one’s illness-related symptoms, requiring a competent level of cognition and adequate health literacy [30]. Dracup and others [31] found low health literacy and global cognition impairment were prevalent in rural HF patients [31]. Second, the SM guidelines, especially those related to sodium restriction and exercise intensity, were found unachievable by many participants [25]. Our data analysis showed that only 5 % HF patients fully adhered to the sodium restriction guideline (≤1500 mg per day). BNP and sodium intake are highly correlated [32]. Without fully complying with the sodium restriction guideline, it would be highly unlikely to observe significant changes in clinical biomarkers (BNP or urine sodium/ creatinine ratio). Similarly, only 6 % HF patients followed the physical activity guideline (≥120 min per week moderate or above intense activity). As a result, adherence behaviors didn’t reach the threshold necessary to have affected the clinical outcomes. Third, our patient sample had much higher numbers of comorbidities compared to other HF populations (8 vs. 3) [22], which could create competing SM demands and further strain SM capability [33]. Last, one explanation for the increased 30-day readmission in the intervention group could be the poorer cardiac functioning in the intervention grouppatients, which could potentially increase risk of 30-day readmission [34]. However, even within class III patients only, readmission rates were higher in the intervention group (28 %) than in the usual care group (0 %). Another potential explanation is the HF patients who received the PATCH intervention might have had improved knowledge and skills in recognizing warning signs of HF exacerbation, communicating their health concerns to their providers, leading to appropriate hospitalization to prevent complications. Dracup also reported higher number of healthcare use in the intervention group. She suggested that the HF patients in the intervention group were educated to pay closer attention to their symptoms, leading to subsequent hospitalizations [24]. Whether this patient-initiated hospitalization improved the health outcomes or not was not examined due to short-term follow up (6 months) in our study.

### Limitations and challenges

Several limitations exist in this study. First, the use of convenience sampling affects the generalizability of the findings to other HF populations. Second, the small sample size might preclude the detection of significant benefit brought by the intervention, although the small effect sizes associated with the non-significant effects indicate that this was unlikely. Third, participant recruitment may have resulted in selection bias. Patients’ personal preferences, beliefs and experiences play a major role in their decision whether to participate in a clinical study. Often, those at risk (e.g., those with lower literacy, more comorbidity, poorer health status, lack of support, etc.) are likely to decline to enroll in behavioral intervention trials [35]. If patients who enrolled in this study were more confident and more actively engaged in SM behaviors than patients who declined, the intervention effects could have been diluted. Fourth, the intervention was tailored to patients’ activation levels, but we failed to recognize variations in patient SM capability. Based on recommendations from patients, family, clinicians and our own experience, it is important to develop a reliable and practice tool to assess SM capability, then tailor the interventions to SM capability and patient activation level.

### Future research direction

The 30-day readmission has been an important national priority for HF patients given its impact on health outcomes and healthcare cost [36]. However, it may not be an accurate indicator of the effect of SM adherence. In future studies, we plan to extend follow up time to see the impact of patient-initiated hospitalization on long-term health outcomes. In addition, we will also examine the interaction between SM capability and activation level, as well as their impact on SM adherence. The findings may assist clinicians in identifying effective strategies to support SM adherence.

## Conclusion

In summary, results of PATCH trial are consistent with previous trials; the activation-enhancing intervention that improved SM behaviors and adherence without reducing readmissions. Given the epidemic of heart failure burdening on the health care system, we urgently need to identify effective but feasible, sustainable strategies to reduce healthcare utilization and improve quality of life in rural HF populations.

## Abbreviations

AHFKT-V2: Atlanta heart failure knowledge test-version 2
BNP: B-type natriuretic peptide
CAH: Critical access hospital
ED: Emergency department
HF: Heart failure
Na/Cr: Urine sodium/creatinine ratio
PAM: Patient activation measure
PATCH: **P**atient **A**c**T**ivated **C**are at **H**ome
RSCB: Revised self-care behavior.
SM: Self-management
UC: Usual care
